# Significant clinical differences but not outcomes between *Klebsiella aerogenes* and *Enterobacter cloacae* bloodstream infections: a comparative cohort study

**DOI:** 10.1007/s15010-023-02010-1

**Published:** 2023-03-07

**Authors:** Kevin B. Laupland, Felicity Edwards, Patrick N. A. Harris, David L. Paterson

**Affiliations:** 1 Department of Intensive Care Services, Royal Brisbane and Women’s Hospital, Level 3 Ned Hanlon Building, Butterfield Street, Brisbane, QLD 4029 Australia; 2 Queensland University of Technology (QUT), Brisbane, QLD Australia; 3 Faculty of Medicine, University of Queensland, UQ Center for Clinical Research, Brisbane, Australia; 4 Infectious Diseases Unit, Royal Brisbane and Women’s Hospital, Brisbane, Australia; 5 Department of Microbiology, Pathology Queensland, Brisbane, Australia

**Keywords:** Klebsiella, Enterobacter, Incidence, Epidemiology

## Abstract

**Purpose:**

Although *Klebsiella aerogenes* (formerly *Enterobacter aerogenes*) and *Enterobacter cloacae* share many phenotypic characteristics, controversy exists as to whether they cause clinically distinguishable infections. The objective of this study was to determine the comparative incidence, determinants, and outcomes of *K. aerogenes* and *E. cloacae* bloodstream infections (BSI).

**Methods:**

Population-based surveillance was conducted among residents aged ≥ 15 years of Queensland, Australia during 2000–2019.

**Results:**

Overall 695 and 2879 incident *K. aerogenes* and *E. cloacae* BSIs were identified for incidence rates of 1.1 and 4.4 per 100,000 population, respectively. There was a marked increase in incidence associated with older age and with males with both species. Patients with *K. aerogenes* BSIs were older, were more likely male, to have community-associated disease, and to have a genitourinary source of infection. In contrast, *E. cloacae* were more likely to have co-morbid diagnoses of liver disease and malignancy and be associated with antimicrobial resistance. *Enterobacter cloacae* were significantly more likely to have repeat episodes of BSI as compared to *K. aerogenes*. However, no differences in length of stay or all cause 30-day case-fatality were observed.

**Conclusion:**

Although significant demographic and clinical differences exist between *K. aerogenes* and *E. cloacae* BSI, they share similar outcomes.

## Introduction

*Enterobacter* species are important causes of infections both in community and institutional settings [1, 2]. *Enterobacter cloacae* is the most common species causing human disease among the more than twenty species belonging to the genus [1]. As a result of chromosomally encoded AmpC b-lactamases and a propensity to acquire other genes, multi-drug resistant *Enterobacter* species are of significant clinical importance due to their risk for treatment failure and relapse [3–6]. Traditionally, the second most common species within the genus causing human disease was *Enterobacter aerogenes* [1]. However, based on genetic relatedness studies, this organism has been reclassified and renamed as *Klebsiella aerogenes*.

Despite the phenotypic similarities between *Enterobacter cloacae* and *Klebsiella aerogenes*, controversy exists as to whether they cause clinically distinguishable infections and/or result in different outcomes. A number of investigations have been undertaken to examine this question in North America, Europe, and Asia [7–10]. However, these studies were underpowered to detect significant differences due to small sample sizes. In addition, prior investigations were limited by conduct at selected hospital(s) such that they were at risk for several important biases [11]. The objective of this study was therefore to determine the comparative incidence, clinical determinants, and outcomes of *Klebsiella aerogenes* and *Enterobacter cloacae* bloodstream infections (BSI) in a large Australian population.

## Patients and methods

A retrospective population-based laboratory surveillance cohort design was utilized. All residents aged 15 years and older who had BSI due to *K. aerogenes* or *E. cloacae* identified within the publicly funded healthcare system in Queensland, Australia during January 1, 2000, and December 31, 2019, were included. The human research ethics committee at Royal Brisbane and Women’s Hospital approved this study and granted a waiver of individual consent (LNR/2020/QRBW/62494).

Pathology Queensland first identified all blood cultures for *K. aerogenes* (recorded as *E. aerogenes*) or *E. cloacae* during the surveillance period. These included all cultures submitted from community and institutional collection sites state wide within the publicly funded system. Pathology Queensland used the BACT/ALERT^®^ 3D system (bioMérieux, Durham, NC) for blood culture testing throughout the study period except for use of the BACT/ALERT^®^ VIRTUO^®^ system (bioMérieux, Durham, NC) from 2018 and thereafter at the main central laboratory that services Greater Brisbane area and some rural Queensland sites. At Pathology Queensland blood cultures are routinely incubated for a minimum of 5 days. BacT/ALERT FA plus (aerobic) and FN plus (anaerobic) media bottles were used as standard. Methods for species identification through the study period included VITEK^®^2 GN ID (bioMérieux), API 20E (bioMérieux) and MALDI-TOF MS (VITEK MS; bioMérieux). Antibiotic susceptibility testing was performed using both an automated method (i.e., VITEK^®^ AST card) and disc diffusion according to recognized standards (CLSI or EUCAST) at the time of testing.

Admission, clinical, and outcome information was obtained through linkages to state-wide hospital admissions and death registries. Previously validated definitions were applied to classify episodes of BSI [12, 13]. Incident episodes were defined by the first isolation of *K. aerogenes* or *E. cloacae* per patient per 30 days and isolation of these organisms with at least one other species within 48 h defined a polymicrobial infection. Admissions to any private or public institutions within the state were identified and discharge diagnostic codes (ICD-10AM) were obtained. Contiguous admissions (i.e., transfer between institutions) were deemed to represent a single admission episode. Deaths occurring in any location within the state on or before December 31, 2020, were identified using the Registry of General Deaths. Hospital-onset, healthcare-associated, and community-associated BSI were classified as per the method of Friedman et al.[14], and comorbidities were defined as per Charlson et al.[15, 16]. A clinical focus was assigned based on review of diagnosis-related group and primary diagnosis hospital discharge codes.

Analysis was performed using Stata 17 (StataCorp, College Station, USA). Incident BSI episodes were age- and sex-standardized (to 2019 Queensland population) using 5-year strata and reported as rates per 100,000 residents [17]. Incidence rates were compared using incidence rate ratios (IRR) with exact 95% confidence intervals (CI). Skewed continuous variables were described using medians with interquartile ranges (IQR) and groups were compared using the Mann–Whitney–Wilcoxon test. Categorical data were compared using Fisher’s exact test. A multivariable logistic regression model was developed to examine factors associated with all cause 30-days case fatality. Age, sex, onset classification, antimicrobial resistance, Charlson Comorbidity Index, polymicrobial infection, and focus of infection were included in the initial model. Stepwise backward variable elimination was performed to develop the most parsimonious model. Calibration and discrimination were assessed using the Hosmer–Lemeshow test and the area under the receiver operator characteristic curve, respectively. *P* values < 0.05 were deemed to represent statistical significance.

## Results

During the two decades of surveillance, 695 and 2879 incident *K. aerogenes* and *E. cloacae* BSIs were identified for annual sex- and age-standardized incidence rates of 1.1 and 4.4 per 100,000 population, respectively. No significant differences were observed between *K. aerogenes* and *E. cloacae* in the distribution of incident cases by month or year of study or by region within the state.

### Demographic determinants

**Fig. 1:**
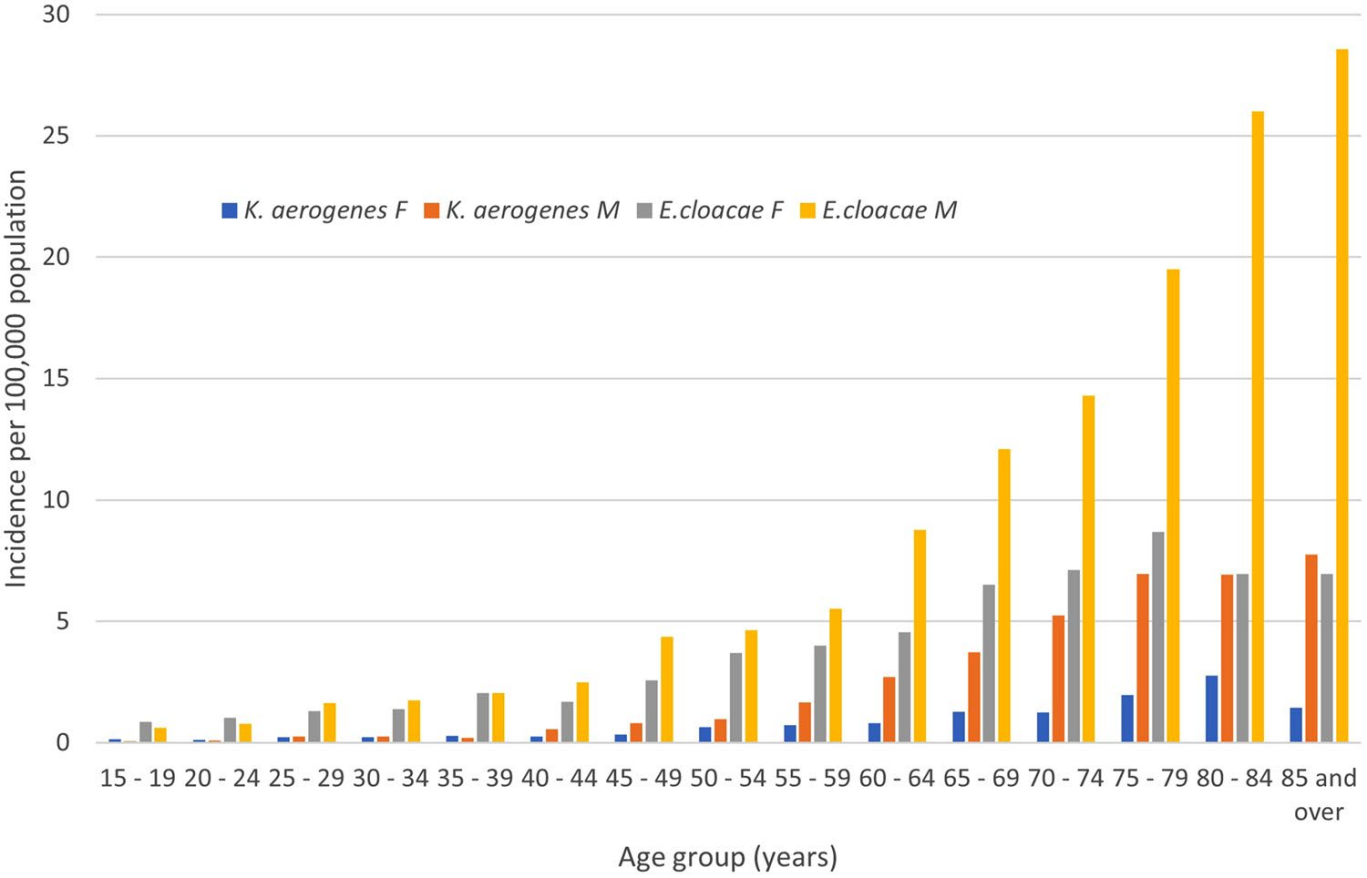
Age- and sex-specific incidence rates of *Klebsiella aerogenes* and *Enterobacter cloacae* bloodstream infections (F = female; M = male)

The median age (IQR) of *K. aerogenes* cases was 67.1 (56.6–76.7) years and this was significantly older than that of patients with *E. cloacae* BSI (median 63.8, IQR 49.5–75.0; *p* < 0.001). A higher proportion of *K. aerogenes* BSI cases were male (493/695; 70.9%) as compared to those with *E. cloacae* (1805/2879; 62.7%; *p* < 0.001). The age and sex distribution of BSI episodes showed a marked increase in incidence associated with older age with both species as shown in Fig. 1. Overall males were at increased risk for BSI both with *K. aerogenes* (IRR 2.49; 95% CI 2.11–2.95; *p* < 0.001) and *E. cloacae* (IRR 1.71; 95% CI 1.59–1.85; *p* < 0.001). However, this significant sex-related excess risk was observed only in those aged 40 years and older for both *K. aerogenes* (IRR 2.84; 95% CI 2.38–3.40; *p* < 0.001) and *E. cloacae* (IRR 1.91; 95% CI 1.76–2.08; *p* < 0.001). Furthermore, while the excess risk in males was relatively constant with increasing age among those with *K. aerogenes* BSI, the excess risk continued to increase in magnitude through to the oldest group of those with *E. cloacae* BSI (Fig. 1).

### Clinical determinants and microbiology

There were several clinical determinants that differed among the two species, and these are displayed in Table 1. While the proportion of community-onset cases was not different between species, exposure to healthcare was significantly (*p* < 0.001) more common among *E. cloacae* cases with 2311 (80.3%) being either hospital onset or healthcare associated as compared to 505 (72.6%) for *K. aerogenes*. The presence of co-morbid diseases was similar with the exception that both liver disease and malignancy was higher among *E. cloacae* as compared to *K. aerogenes* BSI (Table 1). The distribution of focus of infection was different between the species and this was attributable to a higher proportion of non-focal (*p* = 0.004) and a lower proportion of pelvic/genitourinary foci (*p* < 0.001) observed among *E. cloacae* as compared to *K. aerogenes* BSI. Resistance to antimicrobials was significantly higher among *E. cloacae* isolates (Table 1).

**Table 1:** Clinical factors and microbiology associated with *Klebsiella aerogenes* and *Enterobacter cloacae* bloodstream infection

Factor	*Klebsiella aerogenes* *N* = 695	*Enterobacter cloacae* *N* = 2879	*p* value
Onset classification			< 0.001
Hospital	308 (44.3%)	1346 (46.8%)	
Healthcare associated	197 (28.3%)	965 (33.5%)	
Community associated	190 (27.3%)	568 (19.7%)	
Median Charlson (IQR) score	2 (1–5)	2 (1–5)	0.079
Charlson variables			
Myocardial infarction	79 (11.4%)	277 (9.6%)	0.2
Congestive heart failure	119 (17.1%)	491 (17.1%)	1.0
Peripheral vascular disease	57 (8.2%)	249 (8.7%)	0.8
Cerebrovascular disease	48 (6.9%)	219 (7.6%)	0.6
Dementia	30 (4.3%)	97 (3.3%)	0.3
Chronic pulmonary	87 (12.5%)	316 (11.0%)	0.2
Rheumatic	7 (1.0%)	45 (1.6%)	0.4
Peptic ulcer disease	21 (3.0%)	101 (3.5%)	0.6
Liver disease	59 (8.5%)	354 (12.3%)	0.004
Diabetes mellitus	205 (29.5%)	765 (26.6%)	0.1
Plegia	29 (4.2%)	164 (5.7%)	0.1
Renal disease	150 (21.6%)	675 (23.5%)	0.3
Malignancy	193 (27.7%)	983 (34.1%)	0.001
HIV	1 (0.1%)	9 (0.3%)	0.7
Focus of infection			< 0.001
No focus	393 (56.6%)	1800 (62.5%)	
Soft tissue	18 (2.6%)	126 (4.4%)	
Bone and joint	5 (0.7%)	59 (2.1%)	
Upper respiratory	1 (0.1%)	7 (0.2%)	
Lower respiratory	26 (3.7%)	99 (3.4%)	
Endovascular	6 (0.8%)	42 (1.5%)	
Central nervous	3 (0.4%)	10 (0.4%)	
Intrabdominal	130 (18.7%)	470 (16.3%)	
Genitourinary	113 (16.3%)	266 (9.2%)	
Polymicrobial etiology	154 (22.2%)	712 (24.7%)	0.2
Antimicrobial resistance			
Ciprofloxacin	12/680 (1.8%)	107/2759 (3.9%)	0.005
Co-trimoxazole	8/681 (1.1%)	492/2790 (17.6%)	< 0.001
Gentamicin	2/695 (0.3%)	228/2879 (8.1%)	< 0.001
Tobramycin	2/679 (0.3%)	202/2781 (7.3%)	< 0.001
Ceftriaxone	140/601 (23.3%)	605/2487 (24.3%)	0.6
Meropenem	1/638 (0.1%)	23/2555 (0.9%)	0.07
Piperacillin-tazobactam	396/533 (25.3%)	446/2193 (20.3%)	< 0.001
Cefepime	4/544 (0.7%)	166/2246 (7.4%)	< 0.001

### Hospital course and outcome

The overall lengths of stay among 687 (98.8%) and 2838 (98.6%) patients admitted to hospital with *K. aerogenes* and *E. cloacae* BSI were not significantly different (*p* = 0.07) with medians of 13 (IQR 7–31) and 15 (IQR 8–37) days, respectively.

During the surveillance period, seven (1.0%) patients with *K. aerogenes* had a second episode of incident BSI and this occurred a median of 376 (IQR 42–434) days after the index case. In contrast, among patients with *E. cloacae* BSI, 101 (3.5%) had second, 10 (0.4%) had third, and one had fourth incident episodes; as compared to *K. aerogenes*, *E. cloacae* were almost four-fold higher risk for one or more recurrences (relative risk 3.59; 95% CI 1.68–7.68; *p* < 0.001). The median time between episodes was 106 (56–298) days overall, and 106 (58–297) for first repeat episodes which was not significantly (*p* = 0.5) different for that of *K. aerogenes*.

A total of 94 and 392 patients died within 30-days of *K. aerogenes* and *E. cloacae* BSIs death for all cause case-fatality rates of 13.5% and 13.6% (*p* = 1.0), respectively. All cause 30-days case-fatality was not significantly different between species when limited to mono-microbial infections (57/541; 10.5% versus 284/2167; 13.1%; *p* = 0.1) or among first episodes (93/688; 13.5% versus 373/2767; 13.5%; *p* = 1.0). After adjustment for confounding variables in a logistic regression model (*n* = 3574, goodness of fit *p* = 1.0, area under receiver operator characteristic = 0.7392), no difference between *K. aerogenes* and *E. cloacae* was observed in risk for death as shown in Table 2.

**Table 2:** Logistic regression modeling of factors associated with 30-day all cause case fatality

Factor	Odds ratio	95% confidence interval	*p* value
*Enterobacter cloacae* (versus *Klebsiella aerogenes*)	1.01	0.78–1.30	0.95
Charlson comorbidity index (per point)	1.23	1.18–1.28	< 0.001
Onset classification			
Hospital-onset	1 (ref)	–	
Healthcare-associated	0.76	0.61–0.95	0.18
Community-associated	0.58	0.42–0.80	0.001
Age (per year)	1.03	1.02–1.04	< 0.001
Ceftriaxone resistance	1.55	1.23–1.94	< 0.001
Focus of infection			
No focus	1 (ref)	–	
Lower respiratory	1.80	1.13–2.88	0.014
Genitourinary	0.46	0.30–0.70	< 0.001
Other	0.80	0.62–1.03	0.08

## Discussion

In this study, we report novel data on the comparative incidence, determinants, and outcome associated with *K. aerogenes* and *E. cloacae* BSIs in a large Australian population. We find that *E. cloacae* had a fourfold higher incidence, and that demographic and clinical features and resistance rates are significantly different between these species. However, despite these important differences, these species share similar case-fatality. This study provides compelling evidence that *K. aerogenes* and *E. cloacae* are epidemiologically distinct and result in a different spectrum of clinical illness.

There is a paucity of studies that have reported on the population epidemiology of *Enterobacter* species infections. Al-Hasan et al. examined temporal trends among 38 mono-microbial *Enterobacter* species BSI in Olmsted County, USA, during 1998–2007 and found an incidence of 3.3 per 100,000 population [18]. Of these, 26 and 10 BSI cases were due to *E. cloacae* and *K. aerogenes* for respective incidence rates of 2.2 and 0.9 per 100,000 population [18]. Stokes et al. reported on *E. cloacae* complex BSI in Calgary, Canada, during 2015–2017 and identified 154 isolates corresponding to an annual incidence of 1.2–1.5 per 100,000 population [2]. Other population-based studies investigating a range of pathogens have identified *Enterobacter* species ranking among the top 10 most frequent causes of BSI [19, 20]. We are unaware of prior population-based studies that have specifically examined the epidemiology of *K. aerogenes* BSI.

Studies that have compared the clinical determinants and outcome between *E. cloacae* and *K. aerogenes* BSIs have reported conflicting results [7–10]. Jeon et al. conducted a retrospective, single centre, matched study of 194 patients at a tertiary centre in the Republic of Korea and found that *E. cloacae* complex BSI were at twice the risk for 30-day case-fatality as compared to *K. aerogenes* [7]. Alvarez-Marin conducted a 3-years study in five Spanish hospitals including a total of 285 BSI cases and found that *E. cloacae* BSI (*n* = 196) was associated with a higher co-morbid illness burden than with *K. aerogenes* (*n* = 89) [8]. However, they observed no differences in demographics, acquisition type, source, antimicrobial resistance, or case fatality [8]. Wesevich identified 150 BSI cases over a 14-year period at an academic tertiary care centre in the USA and found no differences in hospital case-fatality between species [9]. However, *K. aerogenes* BSI had a worse outcome as compared to *E. cloacae* when a composite outcome measure of hospital case-fatality, recurrence, or complication was analyzed [9]. Song et al. examined 239 BSI cases in the Republic of Korea and found higher rates of resistance with *E. cloacae* (*n* = 172) infections, although *K aerogenes* (*n* = 67) was associated with more severe disease and a worse outcome [10].

We did not observe any differences in all cause case fatality among BSIs due to *K. aerogenes* and *E. cloacae*., and this was true for both crude and adjusted analyses. It is notable that ceftriaxone resistance was a significant independent variable associated with death (Table 2). We previously conducted a study in one Queensland and three New South Wales hospitals and found that relapsing or persistent *Enterobacter* species bacteremia between 3 and 28 days post index culture was infrequent and that emergence of resistance to third generation cephalosporins was low [3]. There remains considerable debate as to whether cephalosporins or b-lactam b-lactamase inhibitor combination agents may be used to treat *Enterobacter* species infections [3, 21–23].

There are some strengths and limitations of our study that merit discussion. Our study cohort was approximately tenfold larger than any previous investigations comparing *K. aerogenes* and *E. cloacae* BSI [7–10]. As a result, we had higher statistical power to detect differences between these species. Another design advantage is that we included all cases identified within the publicly funded system state-wide, such that biases associated with study of selected hospital(s), including referral bias, were minimized [11, 24]. However, it is a limitation that we did not include private laboratories in our surveillance. While we suspect that these represent a limited proportion of cases, we are unable to quantify this potential bias. Our study was retrospective, and as a result we were limited to previously recorded data available in existing databases. Variables such as antimicrobial treatments and severity of illness scores were not available.

In summary, this study is a major addition to the body of literature. We highlight the different clinical features and epidemiology between *E. cloacae* and *K. aerogenes* BSI, and by study of a large cohort demonstrate that these species share a similar outcome.

## Data Availability

Data cannot be shared publicly due to institutional ethics, privacy, and confidentiality regulations. Data release for the purposes of research under section 280 of the Public Health Act 2005 requires application to the Director General (PHA@health.qld.gov.au).
